# Status of disease prevalence, drugs and antibiotics usage in pond-based aquaculture at Narsingdi district, Bangladesh: A major public health concern and strategic appraisal for mitigation

**DOI:** 10.1016/j.heliyon.2022.e09060

**Published:** 2022-03-04

**Authors:** Md. Abu Kawsar, Md. Tariqul Alam, Debasish Pandit, Md. Moshiur Rahman, Mamun Mia, Anuradha Talukdar, Tofael Ahmed Sumon

**Affiliations:** a Department of Aquaculture, Sylhet Agricultural University, Sylhet, Bangladesh; b Department of Aquatic Resource Management, Sylhet Agricultural University, Sylhet, Bangladesh; c Department of Fish Health Management, Sylhet Agricultural University, Sylhet, Bangladesh; d Department of Coastal and Marine Fisheries, Sylhet Agricultural University, Sylhet, Bangladesh

**Keywords:** Antibiotics, Antimicrobial resistance, Fish health management, Probiotics

## Abstract

This research aimed to investigate the present status of disease prevalence and usage of aqua drugs for various aquaculture operations in the Narsingdi region of Bangladesh. Data were collected through the market survey, preset questionnaire interview, personal contact, and participatory rural appraisal tools. Amongst the respondents, the maximum percentages were found practicing mixed cultures of carp, tilapia, and pangas. The respondents suggested that epizootic ulcerative syndrome, saprolegniasis, streptococcosis, tail and fin rot and bacillary necrosis are common fish diseases in the area. About 140 drugs of different companies used in aquaculture for different purposes such as disease treatment, growth enhancement, water quality improvement, toxic gas removal, improvement of feed conversion ratio. Zeolite, rotenone, disinfectant, oxygen precursors, ammonia reducers, and probiotics were applied for pond preparation, water, and soil quality maintenance, while 30 different antibiotics were used for the purpose of treatment. Among the available antibiotics, oxytetracycline, ciprofloxacin, enrofloxacin, erythromycin, sulphadiazine, and trimethoprim were found extensively used by the fish farmers. Four enzymes and eighteen growth promoters were identified as being utilized to enhance digestion and boost up the production. This study elicited various issues connected with application and administration of such aqua chemicals, including farmers’ ignorance about their usage, proper doses, application methods, withdrawal period, and the human health concerns associated with their irresponsible use. However, the consequences of these chemical products to the environment, animal health, and human health required further study for the betterment of mankind.

## Highlights

• EUS, saprolegniasis, streptococcosis are the most prevalent disease in Narsingdi district.
• Around 140 aqua drugs and chemicals are currently in use for treatment of those diseases.
• About 40 different antibiotics are found in use by the farm managers.
• A proper education of using drugs and chemicals is recommended for the farmers.

## 1 Introduction

Aquaculture already accounts for 62.5% of the world's fish production for human consumption (FAO, 2018). Bangladesh is now one of the world's major aquaculture producers, with an annual production of 2.58 million metric tons in 2019-20, and standing as the fifth leading inland aquaculture producer globally (DoF, 2020). This sector (both culture and capture fishery) of Bangladesh produces 4.5 million metric tons of fish, contributing 3.52 % and 26.37% of the national and agricultural GDP (Gross Domestic Product), respectively. More than 12 % of people of Bangladesh are involved in fisheries and aquaculture activities on a full- and part-time basis for their subsistence (DoF, 2019). Bangladesh's aquaculture sector is expanding since the state's inland output is only marginally lower than China's. Indeed, the industry serves as a second source of export revenue for the government (Shamsuzzaman et al., 2017, 2020). The Aquaculture industry has expanded, varied, increased, and mechanically commendable in Bangladesh over the last decades. Chemicals are, in fact, the critical components of thriving farming and have been utilized in numerous structures over centuries (Faruk et al., 2008). Aqua drugs are crucial for aquatic animal health management, pond installation, water and soil quality management, feed formulation, enrichment of natural production, reproductive manipulation, live fish transportation, growth stimulation, processing, and end-product value addition (Subasinghe et al., 1996; GESAMP, 1997).

Apart from the massive use of antibiotics, aquaculture uses a variety of medicines to keep fish healthy and produce more. Potassium permanganate, sodium chloride, malachite green, formalin, glutaraldehyde, methylene blue, and hydrogen peroxide are the most frequently utilized compounds (Plumb, 1992; Sumon et al., 2020). Sodium chloride is a traditional remedy especially for treating fungal and parasitic infections in fish (Phillips, 1996). *Formalin* is a versatile chemical that is used to treat fungal infections as well as flush fish and fish eggs in hatcheries. Potassium permanganate (KMnO4) is the powerful oxidizer that's been certified for use in ponds for external bacterial and protozoan infestations on skin, gills, and fins (Floyd, 1993; Plumb, 1992). Antibiotics were used in aquaculture for more than 50 years to treat bacterial infections in fish (Shamsuzzaman and Biswas, 2012). Recent research has shown that antibiotic use in aquaculture and the aquatic ecosystem has been linked to the development of antimicrobial resistance (Rahman et al., 2009). Pesticides like organophosphates, rotenone, and saponin are also employed to treat disease in aquaculture. Trichlorfon, melalhion, and diptarex are the most often used organophosphates in finfish aquaculture to prevent ectoparasitic crustacean infestation. Organophosphates' substantial neurotoxicity can have substantial negative impacts on the health safety of fish farm laborers (Alderman et al., 1994).

In Bangladesh, farmed aquatic animals were found to be infected with a variety of diseases (Karim and Stellwagen, 1998; Faruk et al., 2004; BFRI, 1999). Due to a lack of vaccination and good health management practices, disease problems in Bangladesh's aquaculture business remain unresolved. Experts advised that the fish vaccinations for disease resistance prevent death and economic loss (Assefa and Abunna, 2018), but in this region, this vaccination initiative needs a massive process, infrastructure, funding, and timeline (Asif et al., 2021). To combat fish diseases, farmers apply a variety of chemicals and medicines. Regrettably, monitoring drugs and antibiotics used in the country's aquaculture industry has received only a little attention, resulting in the aquaculture sector suffering. Farmers have been urged by chemists and representatives from various pharmaceutical companies to use their medicines on this occasion. However, the majority of the farmers indiscriminately apply such chemicals without understanding their requirements, efficacies, and method of administration and considering this issue; the current study was carried out to assess the status of disease prevalence and to identify the different types of chemicals and antibiotics used in the health management of fish and their purposes, dosages with potential concerns.

## 2 Materials and methods

**Figure 1:**
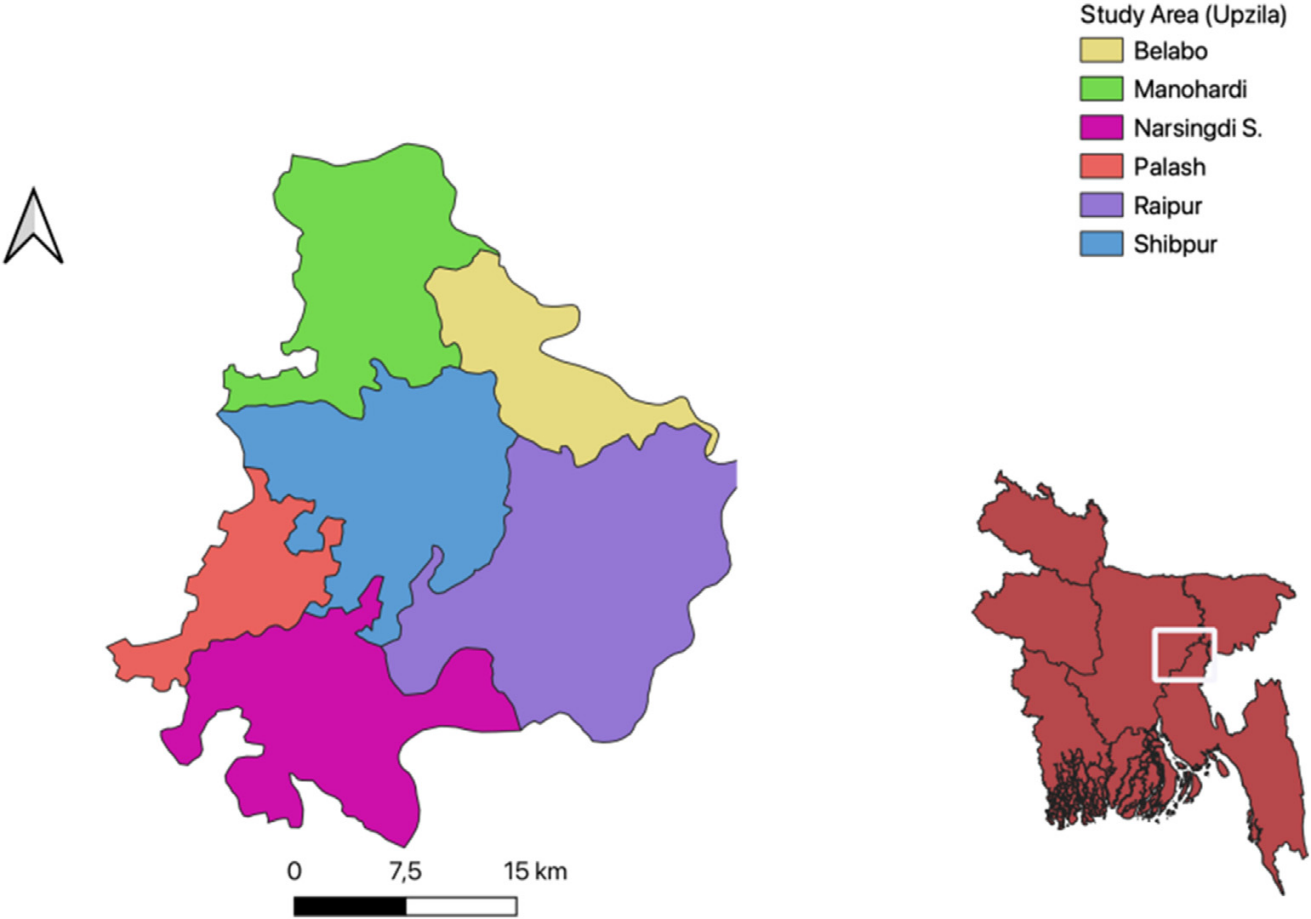
Map showing study areas in Narsingdi district. A total of 6 upazilas i.e., Belabo, Monohardi, Narsingdi South, Palash, Raipur and Shibpur were investigated.

The survey was undertaken randomly in 3 hatcheries, 7 nurseries, 90 grow-out farms, 20 medicine outlets, and 10 aquaculture specialists from various fish feed and pharmaceutical industries from January to December 2020 in the Narsingdi district of Bangladesh (Figure 1). The study area was selected because the Narsingdi district is well-known for its semi-intensive commercial fish farming, and no study on this issue has been conducted in this area. Properly structured questionnaires were designed and pre-tested by several farmers in the surrounding areas before collecting the primary data. The questionnaire focused mainly on the status of fish farming systems, diseases prevalence, affected species and therapeutants they used. Fish diseases were identified based on physical appearance and farmer's observation about infected fish. Through photographs of clinical signs, the interviewer helped them in identifying the diseases. During pre-testing, all essential information regarding the objective's completion was given much thought. Primary data were collected by a survey of farmer households in selected sites after the final questionnaire was improved, using participatory rural appraisal techniques like direct farmer interviews, personal contact, focus group discussions, and crosschecks. Several focus group discussions were held in 6 Upazilas (sub-districts) within the Narsingdi region, with each group having 15 and 25 members. The district fisheries office, upazila fisheries office, private aquaculture professionals, and medicine shops owner provided valuable secondary data as well as the available previous literatures were reviewed extensivelywhich includes online scientific articles, government surveys, books, international studies, and media reports on aqua drugs and antibiotics used in land-based aquaculture.

The study subsequently compiled, accumulated, and analyzed all the data in MS Excel version 2010 and represented in tabular and descriptive statistical techniques.

### 2.1 Ethical statement

All procedures performed in studies involving animals (fish) were in accordance with the ethical standards of the “Sylhet Agricultural University Ethical Committee”. Informed consent was obtained from all individual participants included in the study.

## 3 Results

### 3.1 Health management tools and culture strategies

**Figure 2:**
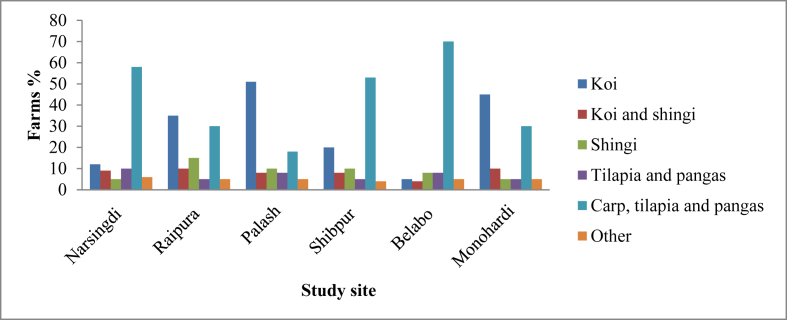
Categories of fish cultured in the study area.

The studied farms in this experiment are assigned into six categories. Such as, polyculture of carp, tilapia and pangas, polyculture of tilapia and pangas, polyculture of koi and shingi, mono culture of shingi, koi and all others were categorized as others (Figure 2). In carps, monosex tilapia, and pangas polyculture, the maximum percentages were found at Belabo (∼70%), Shibpur (∼60%) and Narsingdi (∼60%). The significant number of Vietnamese koi monoculture was recorded in Palash, Monohardi, and Raipura with a percentage of about 50, 45 and 35 respectively. Stocking density and culture duration were found varied with culture strategies. Almost all culture system had a duration of 3–6 months and used commercially manufactured feed. It was found that the stocking density of carp, tilapia and pangas were 60,540 fry/ha, monoculture of koi were 748803 fry/ha, monoculture of shingi with 741315 fry/ha, mixed culture of koi and shingi with 864867 fry/ha, and mixed culture of tilapia and pangas had 56834 fry/ha.

### 3.2 Major aqua drugs used in pond preparation

**Figure 3:**
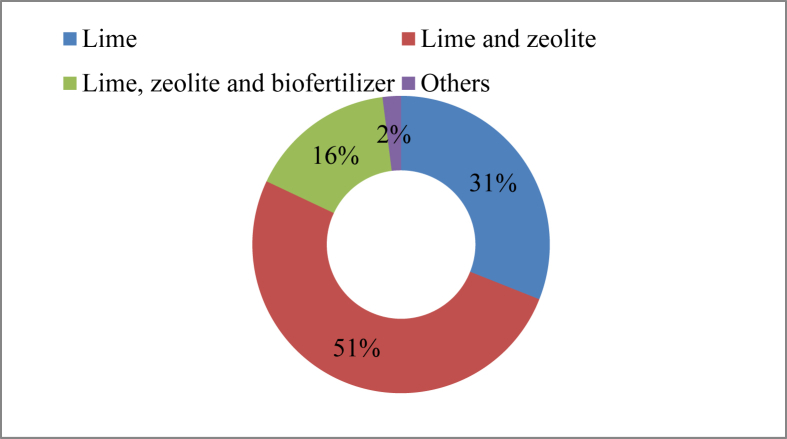
Drugs and chemicals used (%) for pond preparation.

For pond preparation and improvement of the water quality of culture ponds, a variety of conventional and emerging chemical compounds are available on the market. For pond preparation, chemicals like zeolite, lime, biofertilizer and rotenone were applied by the farmers. Table 1 provides a list of the dose suggested by manufacturer and supplier information, of these compounds, along with their active ingredients. Lime and zeolite were found to be the most extensively (51% farmers) (Figure 3) used chemicals. The second highest use of chemicals covered by the application of lime only by 31% of the farmers.

**Table 1:** Chemicals used for pond preparation and water quality management.

Trade name	Active ingredients	Therapeutic class	Dose (Kg/hectare)	Source
Zeofresh	SiO_2_, Al_2_O_3_, Fe_2_O_3_, CaO, MgO, Na_2_O, K_2_O, TiO_2,_	Zeolite	59	Square Pharmaceuticals Ltd (Limited).
ACME's zeolite	SiO_2_, A1_2_O_3_, Fe_2_O_3_, CaO, MgO, Na_2_O	Zeolite	62	The ACME Laboratories Ltd.
Zeopel	SiO_2_-72%, A1_2_O_3_-12%, Fe_2_O_3_-1.9%, CaO-3.7%, MgO-1.2%, K_2_O-3.8%,Na_2_O-0.65%, MnO-0.08%, P_2_O_3_-0.03%, Cr_2_O_3_-0.03%	Zeolite	59	SK + F Pharmaceuticals Ltd.
Zeolite	SiO2, Al2O3, Fe2O3, CaO, MgO, Na2O	Zeolite	62	National Agricare Imp. Exp. Ltd.
Zeo-Ren	SiO2, Al2O3, Fe2O3, CaO, MgO, Na_2_O, K_2_O, P, Mn	Zeolite	62	Renata Ltd.
Zeo prime	SiO_2_-66%, A1_2_O_3_-20%, Fe_2_O_3_-3%, CaO-6%, MgO-3%, K_2_O-3%,Na_2_O-4%, MnO-0.05%, P_2_O_3_-0.16%	Zeolite	59	SK + F Bangladesh Ltd.
JV zeolite	SiO_2_, A1_2_O_3_, Fe_2_O_3_, CaO, MgO, Na_2_O, K_2_O and Mn	Zeolite	54	Eon Animal Health Ltd.
Zeolite gold	SiO_3,_ MgO, CaO_2_ etc.	Zeolite	62	Fishtech BD Ltd.
Geotox	SiO2, Al2O3, Fe2O3, CaO, MgO, Na2O	Zeolite	62	Novartis Animal Health
Mega zeo plus	SiO_2_, A1_2_O_3_, Fe_2_O_3_, CaO, MgO, Na_2_O, K_2_O and Mn	Zeolite	49	ACI Animal Health Ltd.
Lime	CaO, Ca(OH)2		247	Chemical Seller
Aqua lime	CaCO3, Ca(OH)2		247	ACI Animal Health
Matrix	SiO_2_, A1_2_O_3_, Fe_2_O_3_, CaO, MgO, Na_2_O	Sodium alumino silicate	25	Eon Animal Health Ltd.
Aqua pure	SiO_2_ - 60–65%, Al_2_O_3_- 18–22%, CaO- 15–18%, MgO- 2–5%, Fe_2_O_3_- 2–3%, Na_2_O-1-2%	Sodium alumino silicate	25	Square Pharmaceuticals Ltd.
Hunter	Degueline, tephrosin and toxicarol as rotenoids	Rotenon	15	Eon Animal Health Products Ltd.
Aquagreen-G	Sea weed extract, enzyme precursors and micronutrients	Biofertilizer	12	Square Pharmaceuticals Ltd.
Robot	SiO2 - 45–60%, Al2O3- 20–25%, CaO- 15–20%, MgO- 2–5%, Fe2O3- 2–5%, Na2O-1-2%	Zeolite	25	Catapol Bioscience Ltd.
Matrix super	Probiotics, *Yucca* and Zeolite	Zeolite	25	Fish World

### 3.3 Chemicals used as disinfectants

**Figure 4:**
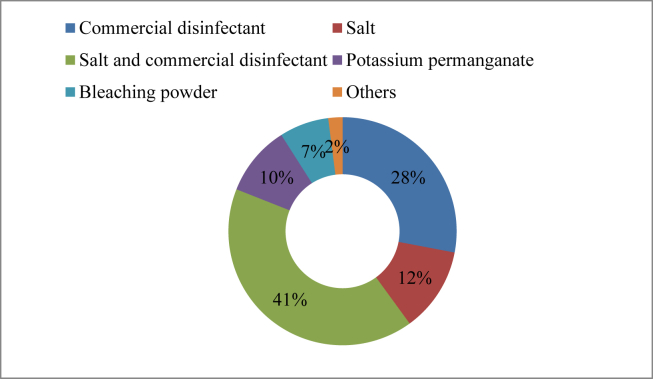
Disinfectants used in the study areas.

In surveyed area, land-based aquaculture operations reported to experience extensive use of disinfectants. According to the company's information leaflet, Timsen, Aquakleen, Sansure, Pathonil, and many other drugs are effective in both preventing and destroying bacterial and fungal diseases, as well as destroying pathogens. Salt, and commercial disinfectant specially BKC (Benzal Konium Chloride) were found to be the most widely used (41%) disinfectant (Figure 4) to treat bacterial and fungal infections. The disinfectants indicated in Table 2 were found in the market. Moreover, some other disinfectants were found in use but with only a small percentage, such as, commercial disinfectant (28%), salt (12%) and potassium permanganate (10%) etc.

**Table 2:** Chemicals used as disinfectant.

Trade name	Active ingredients	Dose	Usages (According to supplier)	Source
Timsen	n-Alkyl dimethyl benzyl ammonium chloride 40%, stabilized urea 60%	0.6 ​kg (kg)/hectare	Preventing agent against viral, bacterial, fungal and parasitic pathogen.	Eon Animal Health Ltd
Aquakleen	tetradecyl trimethyl ammonium bromide: 6.6 g, BKC-83 ​g, amino nitrogen-10000ppm	2.47L L/hectare		Square Pharmaceuticals Ltd
Virex	Potassium peroxymono sulphate 50%	1.49L/hectare		ACI Animal Health
Sansure	BKC-80%	0.74L/hectare		Opsonin Pharma Ltd
Pathonil	Alkyl dimethylbenzyl ammonium chloride 80%, BKC 80%	1.49L/hectare		ACI Animal Health
Polgard plus	3-Methyl and 4-Methyl two chain brominated compound	1.23L/hectare		Fish tech (BD) Ltd.
Micronil	Benzalkonium Chloride 80%	1.48 L/ha		Eskayef pharmaceuticals Ltd.
Povidon aqua	Povidon-Iodine USP	2.47L/hectare		Eskayef pharmaceuticals Ltd.
Povicef	Povidon-Iodine 10%	2.47L/hectare		Opsonin pharmaceuticals Ltd.
Bactrisol-Gold	Alkyl benzul dimethylbenzyl ammonium chloride 80%, BKC 80%	0.74L/hectare		First Agro International
Unidine	Alkyl phenoxy polyglycol ether iodine complex	0.88L/hectare		ACI Animal Health
Potash	KMnO4	2–20 kg/ha		Chemical seller
Salt	NaCl	62–247 kg/hectare		Chemical seller
Bleaching powder	Chlorine	2.47–24.71 kg/ha		Chemical Seller
Pathoside plus	Alkyl benzyl dimethyl benzyl ammonium chloride 80%, Carrier 20%	0.74L/hectare		Fish World
Germidin plus	Iodine 20% with activants	250–500 ml/acre		KRF Agro Care
Farmsafe	Dimethyl benzyl ammonium chloride 5%, *Yucca* extract-q.s, ethanol-q.s,	0.61–1.23L/hectare		Catapol Bioscience Ltd.
GPC 8	Glutaraldehyde patent formula	0.74L/hectare		Reneta Ltd.
Aqua cleaner plus	Kostikthyosulphate, Secondary alken sulphonet, sodium salt, UTDA, Methlium	2.47L/hectare		Fish World
Microbite	Alkyl benzyl dimethyl benzyl ammonium chloride 80%, BKC 80%	0.74L/hectare		Nutrihealth LTD
Aquaxide plus	Alkyl benzyl dimethyl benzyl ammonium chloride 330 ​g, Glutaraldehyde 300 ​g, water q.s.p. 1litre	0.74L/hectare		Advanced Agrotech Ltd.
Virokill aqua	Alkyl benzyl dimethyl benzyl ammonium chloride 80%, water q.s.p....1Litre	0.74L/hectare		Advanced Agrotech Ltd.
Eco safe	Alkyl benzyl dimethyl benzyl ammonium chloride 80%, Excipient q.s.p. 500ml	1.48L/hectare		Nutri Forte Ltd.

### 3.4 Chemical used for oxygen supply

**Figure 5:**
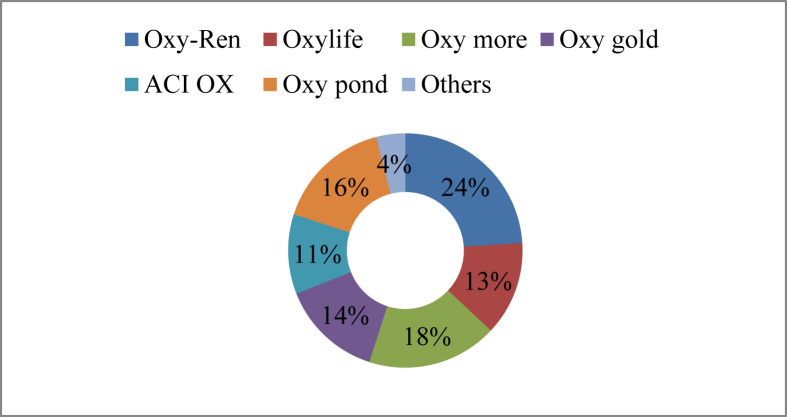
Oxygen enhancers used in the study areas.

The study found several products with identical active ingredients but under different namesin the study area for enhancing oxygen levels in the aquaculture pond. Oxidizing agents, sodium carbonates, and hydrogen peroxide are the main bioactive constituents of those chemicals (Table 3). In the study area, farmers used 12 different oxygen enhancers such as Oxy-Ren (24%), Oxy more (18%), Oxy pond (16%), Oxy gold (16%) and many other brands in their ponds (Figure 5).

**Table 3:** Chemical used for oxygen supply.

Trade name	Active ingredients	Dose	Source
Oxymax	Sodium carbonate, H_2_O_2_	1.23–2.47 ​kg/hectare	Eon animal health products Ltd.
Oxy more	Sodium carbonate per-oxyhydrate	1.23–2.47 ​kg/hectare	SK + F Bangladesh Ltd.
Oxy gold	Sodium percarbonate	1.23–2.47 ​kg/hectare	Fishtech Ltd.
Oxy-A	Sodium percarbonate	1.23–2.47 ​kg/hectare	The Acme Laboratories Ltd.
Best oxygen	Sodium percarbonate	0.61-1.23/hectare	Univet ltd.
Oxylife	Sodium percarbonate	1.23–2.47 ​kg/hectare	Square pharmaceuticals Ltd.
Bio ox	Sodium carbonate,H_2_O_2_	1.23–2.47 ​kg/hectare	ACI animal health
ACI-OX	Sodium carbonate,H_2_O_2_	1.23–2.47 ​kg/hectare	ACI animal health
Oxy flow	Sodium carbonate,H_2_O_2_	1.23–2.47 ​kg/hectare	Elanco Ltd.
Oxy-Ren	Sodium carbonate	1.23–2.47 ​kg/hectare	Renata Ltd.
Oxypond	Sodium percarbonate	1.23–2.47 kg/hectare	Fish world
Oxypol	Sodium percarbonate	1.23–4.94 kg/hectare	Catapol Bioscience Ltd.

### 3.5 Antibiotics for disease treatment

**Figure 6:**
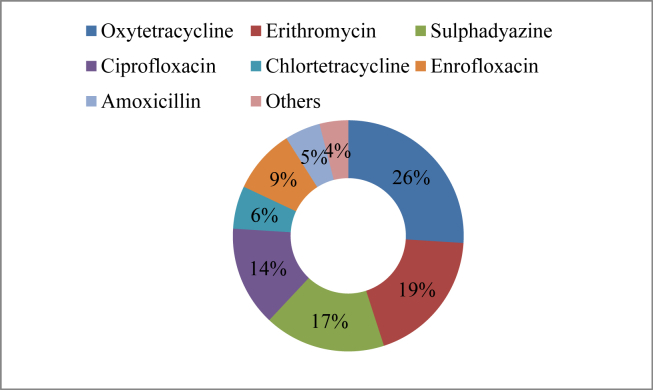
Antibiotics used in the study area.

There were 30 antibiotics found in the study area with various trade names and utilized by fish farmers during the current investigation (Table 4). The active components of these antibiotics are mostly oxytetracycline, chlortetracycline, sulphadiazine trimethoprim, amoxicillin, and sulphamethoxazole. Oxytetracycline (26%), Erythromycin (19%), and Sulphadyazine (17%) were found to be the most commonly used antibiotics, followed by Ciprofloxacin (14%), Enrofloxacin (9%), Chlortetracycline (6%), Amoxicillin (5%), and some other antibiotics with a lower frequency of use (Figure 6). Most farmers are found ignorant about the mode of action of a particular chemical in the current investigation. As a result, while treating a disease, they first test one chemical, and if it doesn't work, they try another. They calculate the dosages of a given chemical based on their own experiences, the instructions on the packet, if any, and the advice of chemical vendors.

**Table 4:** Antibiotics used for disease treatment.

Trade name	Active ingredients	Dose	Source
Otetra-vet 20%	Oxytetracycline	5 gm/kg feed	Square pharmaceuticals Ltd.
Biomycin	Oxytetracycline	5 gm/kg feed	Biopharma Ltd
Aquamycine	Oxytetracycline	5 gm/Kg feed	ACI Animal Health Ltd.
Renamycin	Oxytetracycline	5 gm/kg feed	Renata Ltd.
Oxy-D Vet	Oxytetracycline 20% doxycycline 10%	5-10 gm/kg feed	Eon Animal Health Ltd.
EST-Vet	Erythromycin thiocyanate, Sulphadyazine, trimethoprim	3-5 gm/kg feed	Eon Animal Health Ltd.
Cotrim-vet	Sulphamethoxazole, trimethoprim	5 gm/kg feed	Square pharmaceuticals Ltd.
Sulprim-vet	Sulphadyazine, trimethoprim	3–5 ml/kg feed	Square pharmaceuticals Ltd.
Renatrim	Sulphadyazine, trimethoprim	3–5 ml/kg feed	Renata Ltd
AT-vet	Sulphadyazine, trimethoprim	3–5 ml/kg feed	ACME Laboratories Ltd
Erisen-vet	Erythromycin, sulphadyazine, Trimethoprim	5 gm/kg feed	Square pharmaceuticals Ltd.
Micronid	Erythromycin, sulphadyazine, trimethoprim	5 gm/kg feed	Renata Ltd.
Ciprocin-Vet	Ciprofloxacin	5 ml/kg feed	Square Pharmaceuticals Ltd
Turbonid	Erythromycin, sulphadyazine, trimethoprim	5 gm/kg feed	Eskayef pharmaceuticals Ltd.
Renaquine	Flumequine 20%	3–5 ml/kg feed	Renata Ltd
Levomax	Levofloxacin 10%	5 ml/kg feed	Eskayef pharmaceuticals Ltd.
Maxtor	Chlortetracycline 45%	5 gm/kg feed	Eskayef pharmaceuticals Ltd.
Eska'CTC	Chlortetracycline 20%	5 gm/kg feed	Eskayef pharmaceuticals Ltd.
Enroflox DS	Enrofloxacin BP 20%	3–5 ml/kg feed	Eskayef pharmaceuticals Ltd.
Augment vet	Amoxicillin trihydrate BP& clavulanate BP	5 gm/kg feed	Eskayef pharmaceuticals Ltd.
Ciproflox	Ciprofloxacin 10%	5 ml/kg feed	Eskayef Pharmaceuticals Ltd.
Bactitap	Oxytetracycline hydrochloride	5 gm/kg feed	ACI Animal Health Ltd.
Eryvet	Erythromycin thiocyanate, Sulphadyazine, trimethoprim	5 gm/kg feed	ACI Animal Health
FRA C12	l- Monolaurin & essential oil	5 ml/kg feed	ACI Animal Health
Ciprovet	Ciprofloxacin 10%	5 ml/kg feed	Eon animal health Product Ltd.
Eon CTC	Chlortetracycline 20%	5 gm/kg feed	Eon animal health Product Ltd.
CF-vet-20	Ciprofloxacin	5 gm/kg feed	Prapti Animal Health
Novoflor	Florfenicol	1–2 ml/kg feed	Eskayef Pharmaceuticals Ltd.
Cidaflox	Ciprofloxacin	5 ml/kg feed	Opsonin pharmaceuticals Ltd.
Flumequine	Flumequine BP 20%	5 ml/kg feed	Eon animal health Product Ltd.

### 3.6 Chemicals used as growth promoters

**Figure 7:**
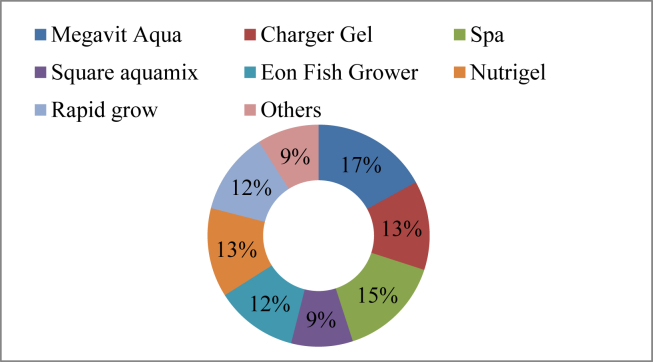
Growth promoters used in the study area.

A variety of chemicals were found available in the market used in aquaculture as growth promoters and production boosters. Most of the growth promoters found in the market contain vitamins, minerals, amino acids, fatty acids, antioxidants, and prebiotics. Similarly, some others were found using for enhancing growth, spawning performance, disease resistance, and bio-availability of the feed supplements (Table 5). Current research revealed that 18 growth promoters were available in the study area. Such as, Megavit Aqua (17%), Spa (15%), Charger Gel and Nutrigel (13%) were the most common brands of growth promoters used by the farmers (Figure 7).

**Table 5:** List of chemicals used as growth promoter.

Trade name	Active ingredients	Dose	Source
Megavit Aqua	Vitamin, mineral and amino acid supplement	1 gm/kg feed	Elanco Ltd.
Charger Gel	1-3 D-Glucan, Polysaccharides, Btain, Beta Glucan	6-8 gm/Kg feed	Fishtech (BD) Ltd.
Aqua bind	Essential amino acid, omega-3 & omega-6 fatty acid	5-15 gm/kg feed	Square Pharmaceuticals Ltd.
Vitamix F aqua	Vitamin, mineral and amino acid	2.5 kg/ton feed	ACME laboratories Ltd.
Acimix super-fish	Vitamin mineral, antioxidant	1 kg/ton feed	ACI Animal Health
Spa	Protein, Cholesterol carotenoid, Vit-D, Ca	10-15 gm/kg feed	Eon Animal Health
Nutrimax	Vitamin, mineral	1 gm/kg feed	SK + F Bangladesh Ltd
Square Aquamix	Vitamins, minerals, and amino acids, prebiotics, yeast, and antioxidant	1 gm/kg feed	Square Pharmaceuticals Ltd
Eon Fish Grower	Vitamin and mineral premix	1.5–3 gm/kg feed	Eon Animal Health Ltd.
Aqua boost	Organic acid and ß-glucan	500 gm/MT feed	Elanco Ltd.
Protifish	18 essential amino acid and minerals	1–5 ml/kg feed	Eskayef pharmaceuticals Ltd
Nutrigel	Feed binder with vitamin, mineral & probiotic	5–10 ml/kg feed	Eskayef pharmaceuticals Ltd
Realbind	Binder with all essential qualities	10 ml/kg feed	Reneta Ltd.
Growth gel	Protein, omega-3 & omega-6 fatty acid, cholesterol, Calcium, Vitamin D3, Carotenoid.	10–15 ml/kg feed	ACI Animal Health
Amino plus	Amino acid, Multivitamins, DCP, probiotics, Trace minerals	3-5 gm/kg feed	Fish World
Vita power	Amino acid and Multivitamin	5 ml/kg feed	Fishtech (BD) Ltd.
Eskalina	Organic spirulina 100%	1-10 gm/kg feed	Eskayef pharmaceuticals Ltd
Rapid grow	Multivitamins	3-5 gm/kg feed	Fishtech (BD) Ltd.

### 3.7 Probiotics used in aquaculture

**Figure 8:**
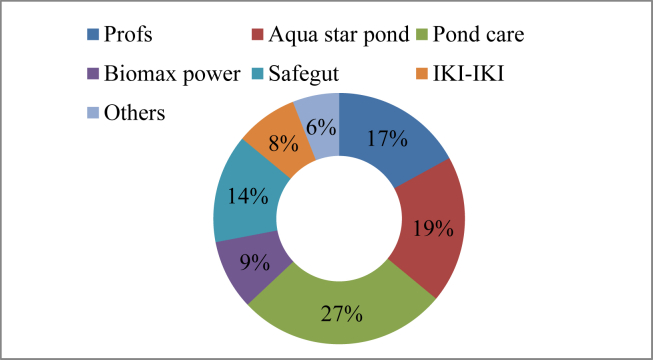
Probiotics used in the study area.

The current study showed that 42% of fish farmers applied 11 different brands of probiotics, such as Pond care (27%), Aqua Star Pond (19%), Profs (17%), and Safegut (14%) and many others (Figure 8) to control disease-causing bacteria, adsorb toxic gases, improve water and soil quality parameters, and promote the proliferation of beneficial microbes. According to respective probiotics companies, they include a variety of beneficial bacteria at different concentrations, such as *Bacillus* sp., *Rhodococcus* sp., *Rodobacter* sp., *Streptococcus faecalis*, and many others (Table 6).

**Table 6:** Probiotics used for fish health management.

Trade name	Active ingredients	Dose	Source
Profs	*Bacillus* sp. and *Padiococcus* sp.	0.37–0.52 ​kg/hectare	Eon Animal Health Ltd.
Bio plus	*Bacillus* sp*. a*nd *Rhodopseudomonas* sp.	3.70–4.94 L/ha	ACI Animal Health
Aqua star pond	*Bacillus* sp*., Pediococcus* sp*., Enterococcus* sp*., Paracoccus* sp. and organic career	0.5–1 kg/ha	Renata Ltd.
Pond care	*Bacillus* sp., *Aspergillus niger* and *Aspergillus oryzae*	0.12 ​kg/hectare	SK + F Bangladesh Ltd.
Biomax power	*Bacillus subtilis* and eight other beneficial bacteria	7.41–9.88 kg/ha	Square Pharmaceuticals Ltd
Protox aqua	*Rhodopseudomonas* sp.	4.94–7.41 L/ha	Square Pharmaceuticals Ltd
Safegut	Lactic acid bacillus, *Bacillus subtilis*, *Bacillus licheniformes*, *Aspergillus oryzae*, *Aspergillus niger*, *Saccharomyces boulardii*, vitamin, and enzyme	3 gm/kg feed	Eskayef pharmaceuticals Ltd.
Aqua photo	*Rhodopseudomonas* sp., *Bacillus subtilis*	4.94–7.41 L/ha	ACI Animal Health
Aquazyme	*Saccharomyces* sp., *Bacillus* sp., Sodium sulphate, Polyvynail alcohol, starch, hydred, selenium, magnesium, and silicate.	0.5–1 gm/kg feed	Eon Animal Health Ltd.
GPA	Multi species probiotics	0.5–1 gm/kg feed	Opsonin pharmaceuticals Ltd.
IKI-IKI	*Bacillus* sp. and *Padiococcus* sp.	0.37 kg/ha	Opsonin pharmaceuticals Ltd.

### 3.8 Chemicals used for obnoxious gas removal

**Figure 9:**
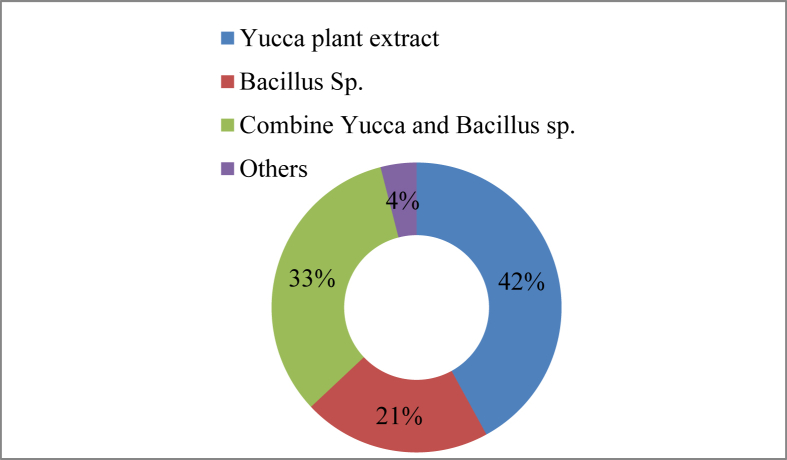
Gas reducers used in the study area.

A variety of toxic gas elimination agents are used by the farmers in their culture ponds. In this study 42% of the farmers used *Yucca* plant extract to remove toxic gas, 33% used a combination of *Yucca* and *Bacillus* sp., 21% just *Bacillus* sp., and 4% used other chemicals (Figure 9). About sixteen toxic gas removers with various trade names were identified (Table 7).

**Table 7:** Chemicals used for toxic gas removal.

Trade name	Active ingredients	Dose	Source
Bio-Aqua-50	*Yucca* plant extract, saponin components glyco components	0.44–0.51L/hectare	Eon animal health
Gasonex plus	Na-lorile ether sulphate	0.49–0.98 kg/ha	Fish tech. (BD) Ltd.
Aqua Magic	*Azotabactor chorococcum, Bacillus subtillis, Candida utilis*	0.98 kg/ha	Fish tech (BD)Ltd.
Gastrap	Lactic acid *Bacillus, Bacillus subtillis* and Enzymes	0.49 kg/ha	Square pharmaceuticals Ltd.
Ammonil	*Yucca* plant extract, *Bacillus subtillis, Candida utilis*	0.24–0.49 kg/ha	Elanco Ltd.
Pondkleen	Extract of *Yucca schidigera*	0.74L/hectare	ACI Animal Health
Ukasol aqua	*Yucca schidigera*	0.74L/hectare	Eskayef pharmaceuticals Ltd
Gasonil	*Bacillus subtillis*, *Bacillus licheniformis*, *Bacillus polymyxa*, *Bacillus coagulan*s, *Yucca* 30%	0.37–0.74 kg/ha	Eskayef pharmaceuticals Ltd
Biopond	SiO2-38_45%, A12O3-33-36%, Fe2O3-1-2%, MgO-0.5%, TiO2-1-2%, FeSO4, MnSO4, Cao, V2O3, CaSO4 and *Bacillus subtillis*, *Bacillus licheniformis*, *Bacillus polymyxa*, *Bacillus megaterium*	2.47–4.94 kg/ha	Eskayef pharmaceuticals Ltd
Aqua4	Zeolite, probiotics, enzyme & yucca	7.41–9.88 kg/ha	Eskayef pharmaceuticals Ltd
Ammo Check	Extract of *Yucca schidigera*	0.74–0.98 L/ha	Navana Phamaceuticals Ltd.
Bio-Aqua plus	Extract of *Yucca schidigera* plant and probiotics	0.49 L/ha	Fish World
First *Yucca* Gold	Extract of *Yucca schidigera*	0.74L/hectare	First Agro International
Gaskit-L	*Yucca schidigera* plant extract, enzyme, *Bacillus subtillis*	0.74L/hectare	CATAPOL bioscience Ltd.
Gaskit-X	*Yucca* and others	0.74L/hectare	CATAPOL bioscience Ltd.
Yuka	Extract of *Yucca schidigera*	0.74L/hectare	Opsonin pharmaceuticals Ltd.
Bioaqua	Extract of *Yucca schidigera*	0.74L/hectare	Nutrihealth LTD

### 3.9 The enzymes used in aquaculture

**Figure 10:**
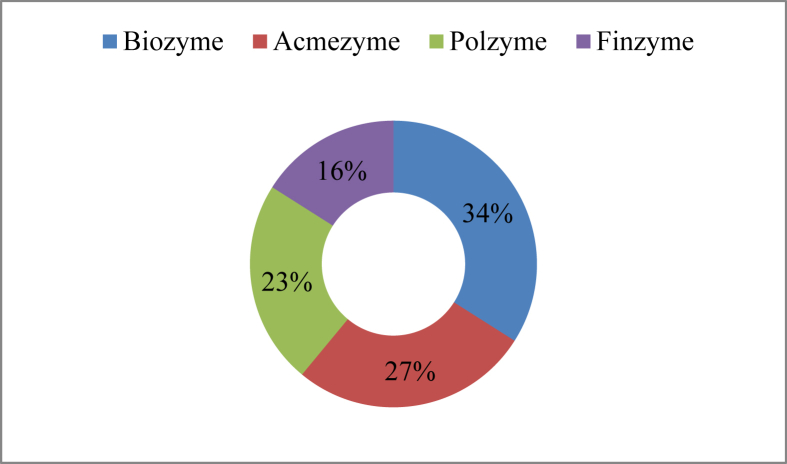
Enzymes used in the study area.

Farmers in the study area applied four types of enzymes (Table 8) *viz*. Biozyme (34%), Acmezyme (27%), Polzyme (23%) and Finzyme (16%) (Figure 10) to boost up the endogenous enzyme activity, dry matter and energy digestibility, growth, survival, intestinal health, and to improve feed conversion ratio.

**Table 8:** Enzymes used in aquaculture.

Trade name	Active ingredients	Dose	Source
Biozyme	Amylase, β-glucanase, lipase, protease, and hemicellulase	0.5gm/kg feed	Fishtech (BD) Limited.
Acmezyme	Cellulase, zylanase, protease, amylase, phytase, pectinase, hemicellulase, and lypase,	1-3 gm/kg feed	ACME Laboratories Ltd.
Polzyme	Protease, cellulase xylanase, lipase, and amylase	1–3 ml/kg feed	Square Pharmaceuticals Ltd.
Finzyme	Cellulase, zylanase, protease, amylase, phytase, pectinase, b-glucanase, and lypase	1-5 gm/kg feed	Eskayef Pharmaceuticals Ltd

### 3.10 Antiparasitic agents used in aquaculture

**Figure 11:**
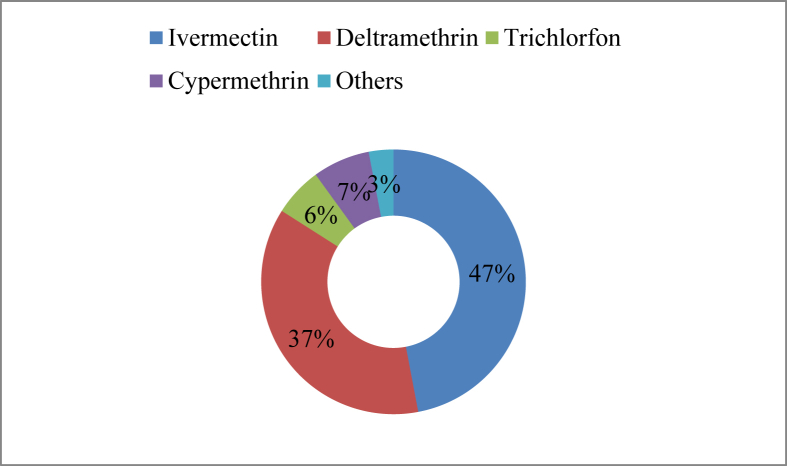
Antiparasitics used in the study area.

The farmers applied eight brands of antiparasitic agents such as Verkil vet, Acimec1% solution, Delitrix etc. in their pond to kill various types of harmful external parasites in the research area (Table 9). The primary active ingredients of the available antiparasitics are ivermectin, deltamethrin, cypermethrin, and trichlorfon which are used by 47%, 37%, 7%, and 6% farmers, respectively (Figure 11).

**Table 9:** Antiparasitics used in aquaculture.

Trade name	Active ingredients	Dose(L/Meter/hectare)	Source
Verkil vet	Ivermectin 1%	0.82	Eskayef Phamaceuticals Ltd.
Acimec1% solution	Ivermectin 1%	2.46	ACI Animal health
Delitrix	Deltramethrin 2.8%	0.41-0.82	Fishtech (BD) Ltd.
Paratrix	Deltramethrin 1.75%	0.41-0.82	Advanced Agrotech Bangladeah
Argulex	Trichlorfon 40%	6.56-8.2	Eon Aquaculture Ltd.
Sumithion	Cypermethrin	4.1–5.74	Sumito Chemical Company Ltd.
First killer	Deltramethrin 2.8%	0.82	First Agro International
Parasite plus	Deltramethrin 2.5%	0.41	Fish World

### 3.11 Common diseases reported

The current investigation identified major clinical signs in diseased fish were red spots and lesions on the body, pop-eyes, abdominal distension, ulcers on the tail, fin, and lower abdominal side. Pop-eyes, reddish ulcers, cotton like fungal growth and abdomen distension were found commonly in Carp, Tilapia, Pangas, koi and shingi. In the study area, the most prevalent diseases were epizootic ulcerative syndrome (90%), saprolegniasis (90%), streptococcosis (80%), fin and tail rot (70%), and bacillary necrosis (60%) with some other diseases being observed with lower incidence (Table 10).

**Table 10:** Prevalence of disease and antimicrobial used in the study area.

Disease		Respondent n = 100	Infected species	Therapeutants they used
Streptococcosis		80%	Tilapia	Erythromycin with commercial disinfectant
Bacillary Necrosis of Pangasius (BNP)		60%	Catfish	Sulphadyazine and trimethoprim with disinfectant
Abdominal dropsy		50%	Tilapia and catfish	Oxytetracycline with enzyme
Fin and Tail Rot		70%	Tilapia, Carp and catfish	Ciprofloxacin with disinfectant
Epizootic Ulcerative Syndrome (EUS)		90%	Carp, tilapia, koi and catfish	Oxytetracycline or amoxicillin with disinfectant
Saprolegniasis		90%	Koi	Amoxicillin with disinfectant
White Spot disease (ICH)		40%	Catfish	Oxytetracycline or ciprofloxacin or enrofloxacin
Lernaeasis		20%	Carp	Ivermectin or deltamethrin with oxytetracycline
Argulosis	30%		Carp	Trichlorfon or Ivermectin with oxytetracycline

## 4 Discussion

Aquaculture is becoming more commercialized and intensive day by day in Bangladesh (Shamsuzzaman et al., 2017). Various kinds of drugs are becoming an essential element of effective aquaculture production. The goal of this study was to learn more about the current scenario of culture compositions, disease prevalence, application of aqua drugs, and their effects on fish health and the environment. There are six types of cultured farms that were investigated in this experiment. The experimental area had the highest percentages of mixed culture of carp, monosex tilapia, and pangas. Some previous research also revealed similar culture compositions in the different areas of Bangladesh (Kawsar et al., 2019; Rahman et al., 2017).

Current investigation showed that farmers applied the nine distinct commercial aqua drugs for various aquaculture operations, especially in health management of fishes. Besides, in pond preparation, disinfecting the culture environment, stimulating growth, and enhancing immunity number of drugs and chemicals were found in use. The primary sources of these chemicals are local animal feed and veterinary medicines stores, which is in the perimeter of the farmers and very easy to purchase. This study identified zeolite and lime as the most used chemical for pond water quality maintenance. In addition lime and zeolites are reportedly a most used chemicals in the area which is consistent to this survey (Chowdhury et al., 2012; Faruk et al., 2008; Kawsar et al., 2019; Shamsuzzaman et al., 2012).

One of the major limitations emerged in aquaculture intensification nowadays is fish diseases, which become a significant constraint in effective and sustainable aquaculture business profitability, in consequences. EUS, fin and tail rot, dropsy, bacillary necrosis, white spot, saprolegniasis, lernaeasis, and argulosis were the most common diseases and symptoms (Table 10) reported by farmers in this study. Several authors have also documented comparable circumstances in Bangladeshi aquaculture industry (Faruk et al., 2004; Amin, 2000; Mazid, 2001). According to the supplier's information, disinfectants and antibiotics were used by farmers to maintain their ponds free of pathogens and to cure several infectious diseases such as Epizootic Ulcerative Syndrome (EUS), Red spot disease, streptococcosis, ichthyophthiriasis, etc. In some cases, the farmer got about 95% recoveries from the disease condition within a short period (Rahman et al., 2017). In the current investigation, 23 disinfectant brands and 30 antibiotic brands with various trade names were found on the market. Similarly, Shamsuzzaman and Biswas (2012) identified 12 disinfectant brands and 14 antibiotic brands on the southwest coast of Bangladesh. The most often used disinfectants in the study area were commercial disinfectant such as BKC and salt potassium permanganate and bleaching powder. Rahman et al. (2017) stated that 22%, Kawsar et al. (2019) claimed that 40% of farmers of the study area used potassium permanganate and 38% were used commercial disinfectant. Antibiotics were found to be administered indiscriminately in the current investigation, although the specific causes of the disease were unknown. Some farmers did not use the prescribed treatment doses. A total of 15 antibiotics were identified and farmers were reported with irresponsible and frequent use of such drugs without approval and without knowing their effects on fish health (Faruk et al., 2021). Several issues regarding improper use of aquatic medications, such as lack of information about chemical use, sufficient dosage, form of application, and indiscriminate use of antibiotics have been reported by Hasan et al. (2020).

Several aqua drugs have been found to be used as oxygen precursors, ammonia reducers, growth promoters, antiparasitics, enzymes, and probiotics to aid digestion and keep the aquatic environment healthy. Antibiotics with six categories of other compounds, including nutritional supplements, disinfectants, saline, ammonia removal, probiotics, and pesticides, were administered by the fish farmers of Mymensingh (Faruk et al., 2021). Previously several authors found very similar outcomes in their research on aqua medicines in Bangladesh (Kawsar et al., 2019; Rahman et., al., 2017; Shamsuzzaman and Biswas, 2012; Chowdhury et al., 2012; Rahman et al., 2015).

### 4.1 Antibiotic exposure pathway in aquaculture

**Figure 12:**
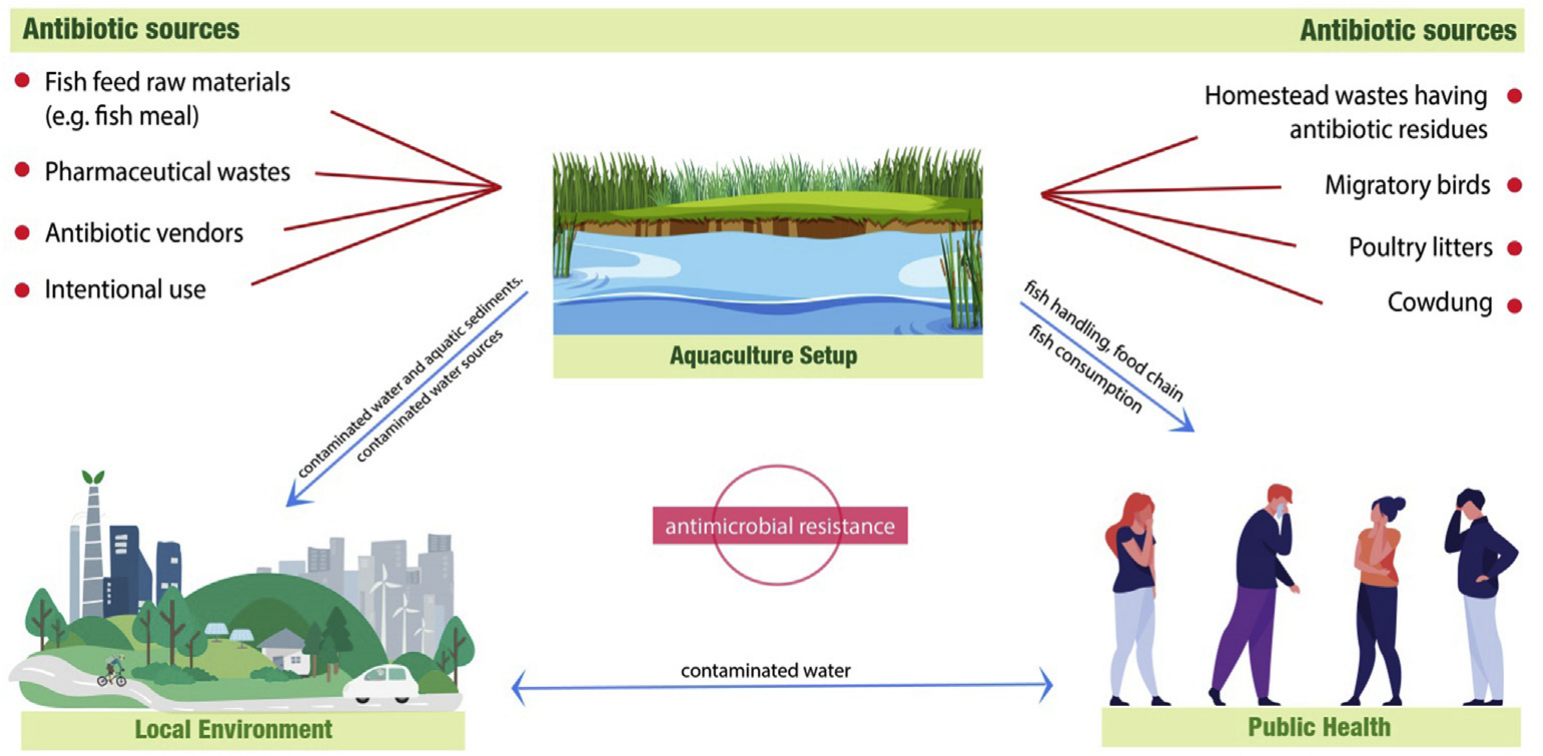
The possible exposure pathway of antibiotics through a hypothetically illustrated aquaculture setup. Different sources like raw materials of the feed, some intentional approaches of the farmers, and various waste disposals input the antibiotics into the setup, which afterward affect the local environment and public health through contaminated water sources and sediments, including consumption and handling of the aquaculture end products and in consequences, antimicrobial resistance becomes developed into different ecological compartments (Santos and Ramos, 2018; Ngogang et al., 2021; Ullah et al., 2020; Thai et al., 2018; Han et al., 2017; Chowdhury et al., 2015; Pruden et al., 2013).

In aquaculture, as in other animal production sectors, similar strategies (e.g., vaccination and use of antibiotics) are used to control infectious diseases. Antimicrobial use in aquaculture differs from cattle farming due to the greater diversity of species, farming practices, and different application methods. The application of antimicrobial in aquaculture ponds has a consequence in the formation of drug-resistant bacteria repositories in aquatic species and even in the ecosystem (Schmidt et al., 2000; Akinbowale et al., 2006; Hamom et al., 2020). Antimicrobials, in whatever form these are applied in food production, these will have significant complications on human health, surrounding environment, and aquatic ecosystems (Rasul and Majumdar, 2017; Brunton et al., 2019). According to the pathways analysis (Figure 12), the two most prevalent techniques of administering antimicrobial drugs in aquaculture species are medicated feed and applying antibiotics directly to the water (immersion therapy), both of which require flock treatment of the animals (Heuer et al., 2009). Antibiotics are most often administered to aquatic animals by combining them with specially formulated feed. However, antibiotics are not efficiently metabolized by fish and are mostly execrated in the environment via faces. Seventy-five percent of antibiotics supplied to fish are estimated to be released into the aquatic environment (Burridge et al., 2010). These practices can lead to excessive usage of antimicrobials and strong selective pressure in the aquatic animals and the exposed surroundings. According to the findings of some previous studies, effluent from antibiotic-treated cattle farms (fecal waste) may end up in aquaculture ponds since cow dung is a popular source of raw ingredients in land-based aquaculture (Jha et al., 2004), potentially introducing antibiotics into the aquaculture setting (Sobur et al., 2019; Kabir et al., 2018).

Another source of antibiotics in fish farms in the study area is the dumping of poultry litter and other slaughterhouse wastes in the pond ecosystem to increase primary production. Resulting antibiotics may gain access to the pond environment, and there has recently been an increase in the incidence of food-borne illnesses caused by antibiotic-resistant bacteria (Teuber, 1999). The use of poultry litter as an aquaculture supplement may transmit certain food-borne or zoonotic bacteria and multidrug-resistant bacteria to the aquaculture setup and consequently to the consumer (Aly et al., 2009).

### 4.2 Antimicrobial resistance (AMR) in aquaculture

We found a total of 30 brands of antibiotics were reported from the study area. A total of 58 antibiotics have been recorded from various parts of Bangladesh previously (Asif et al., 2021). Lulijwa et al. (2020) studied antibiotic usage in key aquaculture-producing countries, including Bangladesh, and found that 19 antibiotics are used in Bangladeshi aquaculture. This study showed that antibiotics were applied indiscreetly by fish farmers without identifying the exact causes of fish diseases. Rahman et al. (2017) described similar findings. Antibiotics used inappropriately or irrationally can lead to the development of antibiotic-resistant bacteria (Inglis V. 2000). The longer an antibiotic is exposed to the environment, the higher the chance of resistance developing, and administering these medications into aquatic environments allows them to survive for lengthy periods. Antibiotic residues have severe public health consequences (Table 11), including antibacterial drug resistance, hypersensitivity reactions, mutagenicity, carcinogenicity, bone marrow suppression, teratogenicity, and disruption of normal gut flora (Schar et al., 2020; Okocha et al., 2018; Miranda et al., 2018).

**Table 11:** The literature revealed the potential health effects of active drug residues.

Active substance	Purpose of use	Impact on human health	Literature cited
Antimicrobial agents	To control infectious disease	Increase the number of infections, frequency of treatment failures, and infection severity. Increased risks of AMR genes.	Kruse and Sorum, 1994; Heuer et al. (2009); Okocha et al. (2018); Tomova et al. (2015)
Disinfectants	Routine sanitation and biosecurity	Cancer and reproductive/developmental effects. Irritation in case of skin and eye contact.	Tsitsifli and Kanakoudis (2018), Watterson et al. (2012)
Pesticides	Elimination of undesired species, renovation and/or complete harvesting	Refractory hypotension, congestive heart failure, brain cancer, prostate cancer, pulmonary dysfunction and electrocardiographic abnormalities	Gurjar et al.,2011; Lee et al. (2011); Andersen et al. (2012)
Vitamin premix	Used as feed supplement	Literature is not available	
Oxygen precursors	Provide ample oxygen during oxygen deficit	Literature is not available	

### 4.3 Options for risk management

The most efficient way to manage and control the emergence and expansion of antimicrobial resistance is to minimize the demand for antibacterial treatment (Moges et al., 2014). A federal administrative framework is necessary for antibacterial agent licensing, approval, monitoring, and regulation in all countries where antimicrobial agents are applied in aquatic biota. Production planning should include stocking strategies and management measures to prevent the invasion of germs and the spread of infectious diseases. As Bangladesh is a leading aquaculture producer, the government should place a high priority on the control and monitoring of aquaculture drugs and chemicals at the field level. When it comes to the usage of aqua drugs and antibiotics, there are a few guidelines to follow, including (i) constantly endeavor to enhance the pond ecosystem, (ii) aqua drugs and antibiotics should only be used when it is essential, (iii) use antimicrobials only for bacterial infection, (iv) use an antimicrobial to which the pathogens are susceptible, (v) arrange training on Good Aquaculture Practices (GAP) for farmers, (vi) Aqua chemicals should be handled with caution since they can be harmful to public health, (vii) use appropriate dose, (viii) Minimum use of chemical is the best alternative to reduce adverse effect.

## 5 Conclusion

Considering the tremendous expansion and significance of the aquaculture sector in several areas of Bangladesh, the study sought to ascertain the current state of aquaculture inputs like chemicals and antimicrobial substance use in aquatic animal care and identified several challenges when it comes to using chemicals, including a lack of understanding of the compounds, their unregulated uses, and application methods. Significant measures are required to combat the establishment and spread of indiscriminate chemical usage and antimicrobial resistance in aquaculture. The efficacy of various aqua products in field trials and fish disease diagnosis based on signs, symptoms, and eye assessment may be considered a significant research gap in this study. However, to reduce the detrimental effects of drugs used in aquafarming, government policymakers, fisheries experts, researchers, farmers, entrepreneurs, the pharmaceutical industry, and scientists should collaborate to address the challenges.

## Declarations

### Author contribution statement

Md. Abu Kawsar: Conceived and designed the experiments; Performed the experiments; Analyzed and interpreted the data; Contributed reagents, materials, analysis tools or data; Wrote the paper.

Md. Tariqul Alam, Debasish Pandit, Md. Moshiur Rahman & Anuradha Talukdar: Contributed reagents, materials, analysis tools or data.

Mamun Mia: Performed the experiments.

Tofael Ahmed Sumon: Analyzed and interpreted the data; Wrote the paper.

### Funding statement

This research did not receive any specific grant from funding agencies in the public, commercial, or not-for-profit sectors.

### Data availability statement

Data included in article/supplementary material/referenced in article.

### Declaration of interests statement

The authors declare no conflict of interest.

### Additional information

No additional information is available for this paper.
